# Dr. E. Arnold Praeger

**Published:** 1898-04

**Authors:** 


Dr. E. Arnold Praeger, of Los Angeles, Cal., died at his residence in that city March 6, 1898, aged 43 years. He was professor of gynecology at the Los Angeles Polyclinic, clinical gynecologist at the free dispensary, Fellow of the Obstetrical Society of London, Fellow of the American Association of Obstetricians and Gynecologists, member of the British and Canadian Medical Associations, member of the Southern California and the Los Angeles County Medical Societies. Dr. Praeger was a man of accomplishments and a skilful surgeon. His death in the prime of young manhood is greatly to be regretted.

